# Assessment of tumor volume and density as a measure of the response of advanced hepatocellular carcinoma to sorafenib: Application of automated measurements on computed tomography scans

**DOI:** 10.1002/jgh3.12230

**Published:** 2019-08-02

**Authors:** Junta Yamamichi, Yasunori Kawaguchi, Taiga Otsuka, Shunya Nakashita, Hideaki Mizobe, Yuichiro Eguchi, Shinya Kimura

**Affiliations:** ^1^ Medical Equipment Business Planning Department, Medical Systems Operations Canon Inc. Tokyo Japan; ^2^ Department of Hepatobiliary and Pancreatology Saga‐ken Medical Centre Koseikan Saga Japan; ^3^ Department of Oncology Saga‐ken Medical Centre Koseikan Saga Japan; ^4^ Department of Internal Medicine, Faculty of Medicine Saga University Saga Japan

**Keywords:** density, hepatocellular carcinoma, Response Evaluation Criteria in Solid Tumors, sorafenib, survival, volumetry

## Abstract

**Background and Aim:**

To better predict patient survival, we used automated tumor volume and density measurements to make an objective radiological assessment of the response of advanced hepatocellular carcinoma (HCC) to treatment with sorafenib.

**Methods:**

Patients treated with sorafenib were identified retrospectively. Those who were diagnosed with Child‐Pugh class A liver function, Barcelona‐Clinic Liver Cancer stage C, and Eastern Cooperative Oncology Group performance status grade 0/1 were enrolled (*n* = 22). Reviews of contrast‐enhanced computed tomography images were supported by the automated measurement of lesions using computer software. Treatment responses were assessed using volume and density criteria. Kaplan–Meier methods and multivariate Cox regression analysis were used to evaluate treatment responses and identify the most significant prognostic factors for overall survival (OS).

**Results:**

After patients were dichotomized according to volume and density criteria, the median OS for those with an objective response (OR) (complete response + partial response) was 20.4 months and that for those with a non‐OR (stable disease + progressive disease) was 9.3 months (*P* = 0.009). The best multivariate regression model for survival identified volume and density criteria (OR or non‐OR) as a significant variable, along with baseline alpha‐fetoprotein levels (log‐rank test, *P* = 0.01). No other conventional criteria were identified as significant.

**Conclusions:**

Tumor volume and density assessment using automated lesion measurements may be an objective method of evaluating responses of advanced HCC to treatment with sorafenib.

## Graphical abstract

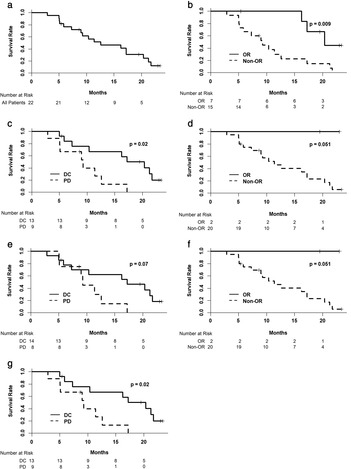

Tumor volume and density assessment using automated lesion measurements may be an objective method of evaluating the responses of advanced hepatocellular carcinoma to treatment with sorafenib.

## Introduction

Primary liver cancer is the sixth most common cancer in the world and the second largest contributor to cancer‐related mortality.^1^ The worldwide incidence of the most common type of cancer, hepatocellular carcinoma (HCC), is growing, and it is estimated that, by 2020, the number of new cases in Europe, the United States, and Japan will reach 70 290, 35 574, and 42 104, respectively.^2^

The oral multityrosine kinase inhibitor sorafenib [Nexavar; Bayer HealthCare Pharmaceuticals (Seattle, WA, USA)–Onyx Pharmaceuticals (South San Francisco, CA, USA)] was the only approved drug that demonstrated survival benefits for patients with advanced unresectable HCC for nearly a decade until the recent approval of regorafenib (Stivarga; Bayer HealthCare Pharmaceuticals; Seattle, WA, USA), used as a second‐line treatment, and lenvatinib (Lenvima, Eisai Co., Ltd, Tokyo, Japan), used as a first‐line treatment.^3^, ^4^, ^5^

Although sorafenib provides patients with HCC with a survival advantage, no study has accomplished a timely and accurate evaluation of its treatment effects. The Response Evaluation Criteria in Solid Tumors (RECIST) guidelines (version 1.1)^6^ may underestimate its efficacy because of its modest ability to shrink tumors. RECIST 1.1 uses unidimensional morphological criteria only. Antiangiogenic agents such as sorafenib induce heterogenic changes in tumor appearance, such as areas of necrosis and irregular changes in the shape of the lesion, by reducing vascularization.^7^, ^8^ Therefore, assessment using RECIST 1.1, which is based on tumor size, raises concerns about its appropriateness as a surrogate end‐point for the survival of patients with HCC.^9^ Thus, alternative response criteria, such as modified RECIST (mRECIST), the European Association for the Study of the Liver (EASL), and the Choi criteria, take into account changes in lesion vascularity or viability, which is measured by contrast‐enhanced computed tomography (CT).^9^ The mRECIST for HCC was adopted by the international guidelines on the management of HCC,^10^ but the use of RECIST1.1 and mRECIST is only suggested for the assessment of response of HCC treated with systemic therapy such as sorafenib because there is no clear evidence of its accuracy.^11^ This might be because these criteria are still dependent on manual radiological assessments based on the simple measurement of the longest diameter (LD) of the lesion.^9^, ^12^ Consequently, three‐dimensional volumetry of tumor masses is proposed as a more reproducible and sensitive method.^13^, ^14^, ^15^, ^16^, ^17^ Indeed, several computer software packages have been developed to assist the taking of objective measurements from CT scans. Previously, we used such software for patients with lung cancer or multiple myeloma^18^, ^19^; these studies demonstrated the efficacy and utility of this software for evaluating responses to cancer treatment in a standardized way. Thus, a large‐scale evaluation of the data collected, and sharing these data in a multicenter clinical trial, was suggested.

Here, we further examined the applicability of a three‐dimensional automated radiological evaluation method based on computer software that can simultaneously measure both the volume and density (attenuation coefficient on CT scan) of target tumors on CT scans. We used this objective approach to evaluate treatment responses of advanced HCC to sorafenib therapy by investigating correlations between survival outcomes and measured lesion parameters.

## Methods

### *Patients*

All consecutive patients with advanced HCC and treated with sorafenib at Saga University Hospital and Saga‐ken Medical Centre Koseikan between July 2008 and March 2012 were identified. Those who were diagnosed with Child‐Pugh class A liver function, Barcelona‐Clinic Liver Cancer (BCLC) stage C, and Eastern Cooperative Oncology Group (ECOG) performance status (PS) grade 0 or 1 were enrolled in this retrospective study. Patients underwent routine practice and so might have received other locoregional treatments before sorafenib treatment. The study protocol was approved by the Clinical Research Ethics Committee at each hospital and complied with the Declaration of Helsinki and its related guidelines.

### *Treatment*

Enrolled patients received sorafenib for at least 30 days. Blood samples were collected at baseline, and pretreatment serum marker levels of alpha‐fetoprotein (AFP), the *Lens culinaris* agglutinin‐reactive fraction of AFP (AFP‐L3%), and Des‐gamma‐carboxy prothrombin (DCP) were measured. Overall survival (OS) was measured from the beginning of sorafenib treatment to the date of death or last follow‐up (right censored).

### *Radiological evaluation of treatment responses*

Contrast‐enhanced spiral CT scans (slice thickness, 5 mm) were performed at baseline (before initiation of treatment) and at every 2–3 months afterward. Computer software was used to help evaluate the best clinical response according to RECIST 1.1 criteria and to allow the automated measurement of lesion volume and density as imaging parameters. In addition, mRECIST assessment was conducted independently from the above measurements as current standard imaging criteria. This study examined the combination of volume and density parameters indicative of better response criteria using automated measurement, but routine evaluations are still widely based on RECIST 1.1 because of its simplicity. Therefore, this study included both RECIST 1.1 and volumetric criteria as a reference to unidimensional measurement and its expansion to three‐dimensional measurement, respectively. Hepatic lesions were measured on contrast‐enhanced images during the arterial phase. Three physicians (Yasunori Kawaguchi, Taiga Otsuka, and Shunya Nakashita) reviewed all images jointly and made a consensus decision about whether a manual correction to a lesion contour was necessary. If a patient was evaluated as having a complete response (CR) or partial response (PR), or as having achieved stable disease (SD), then they were classified as “under disease control (DC).” If a patient showed a CR or PR, they were considered to have an objective response (OR). These evaluations were made at the time of the best clinical response during the treatment course. Tumor density was standardized relative to background liver density. Thus, the density measurements were rendered comparable to better reflect the vascularity of each lesion. For this exploratory study, cut‐off values were determined by taking into account the patients' survival outcome (summarized in Table 1). For progressive disease (PD), they were set as a ≥ 50% increase in tumor volume, while for PR, they were set as a ≥ 50% decrease in tumor volume or a ≥ 15% decrease in tumor density and a ± <50% change in tumor volume. Lesion Management Solutions software (MEDIAN Technologies, Valbonne, France) was used for radiological evaluation. This software supports three‐dimensional quantification and allows the comparison of successive CT scans from the same patient, with synchronous navigation between two scans and automated pairing of lesions.^18^ The software performed automated delineation of the lesion contour and then calculated the volume and density of each lesion.

**Table 1:** Definition of response categories for radiological evaluation criteria

Response	RECIST 1.1	Volumetry	Volume and density†
Complete response	Disappearance of all lesions		
	No new lesion		
Partial response	≥30% decrease in tumor diameter	≥50% decrease in tumor volume	≥50% decrease in tumor volume or ≥15% decrease in tumor density and ±<50% change in tumor volume
	No new lesion	No new lesion	No new lesion
Stable disease	Neither response nor progression		
	No new lesion		
Progressive disease	≥20% increase in tumor diameter or new lesions	≥50% increase in tumor volume or new lesions	

† Tumor density was measured on the late arterial phase acquisition and standardized relative to background liver density.

All measurements are based on the sum of target lesions as defined by Response Evaluation Criteria in Solid Tumors (RECIST) 1.1.^6^

### *Statistical analyses*

Continuous variables are expressed as median or mean values with their ranges, and categorical variables are expressed as numbers and percentages. Median OS time (in months) was estimated using the Kaplan–Meier method. Differences in survival curves between response groups were evaluated using the log‐rank test. Univariate and multivariate Cox regression analyses were performed to identify prognostic factors of OS. Variables with *P* < 0.1 in univariate log‐rank tests were included in multivariate analysis. Selection of the final model was based on Akaike's Information Criterion (AIC). Before conducting Cox regression analyses, the importance of each pretreatment and peritreatment variable was measured using the random forest approach to aid variable selection for entry into Cox regression. Important variables could be critical predictors of survival following sorafenib treatment. AFP and DCP were transformed to a logarithmic scale to reduce the skewness of their distributions.^20^ All statistical analyses were conducted using R version 3.4.0 (The R Foundation for Statistical Computing Platform; Vienna, Austria). A two‐sided significance level of *P* < 0.05 was used for all statistical analyses.

## Results

### *Patient characteristics and treatment*

Initially, 81 consecutive patients treated with sorafenib were identified. Of these, 22 met the inclusion criteria. Their demographic and clinical characteristics are summarized in Table 2. The median age was 76 years (range, 50–86 years). Most patients were male (91%). A majority had a Child‐Pugh score of 5 (64%) and an ECOG PS grade of 0 (86%). The median duration of sorafenib treatment was 2.6 months (range, 1.1–19.5 months). Only one patient was still receiving sorafenib treatment at the end of the follow‐up period. For the other patients, sorafenib treatment was terminated due to tumor progression (68%) or adverse events (27%). The median baseline serum levels of AFP, AFP‐L3%, and DCP were 5215 ng/mL (range, 2.8–48 000 ng/mL), 36.4% (range, 0–91.3%), and 1318 mAU/mL (range, 12–9399 mAU/mL), respectively.

**Table 2:** Patient demographics and clinical characteristics

Variable	*n* = 22 patients
Age (years), Median (Range)	76 (50–86)
Gender	
Male/female	20 (91%)/2 (9%)
Body weight (kg), Median (Range)	55.0 (33.7–92.5)
BMI (kg/m^2^), Median (Range)	21.1 (13.7–30.2)
Etiology	
HBV/HCV/NBNC	4 (18%)/13 (59%)/5 (23%)
Child‐Pugh score	
5/6	14 (64%)/8(36%)
ECOG PS	
0/1	19 (86%)/3 (14%)
Prior treatment	
Yes/no	20 (91%)/2 (9%)
Extrahepatic spread	
Yes/no	13 (59%)/9 (41%)
Vascular invasion	
Yes/no	10 (45%)/12 (55%)
Duration of sorafenib treatment (months), Median (Range)	2.6 (1.1–19.5)
Sorafenib medication status†	
Continued/terminated	1 (5%)/21 (95%)
Reason for sorafenib termination	
Tumor progression/adverse events	15 (71%)/6 (29%)
Post‐treatment	
Yes/no	14 (64%)/8 (36%)
Laboratory tests, Median (Range)	
ALT (IU/L)	40.4 (8–100)
AST (IU/L)	49.0 (17–102)
Total bilirubin (mg/dL)	0.87 (0.5–1.4)
Platelets (/mm^3^)	88.2 (71.6–110.1)
Albumin (g/dL)	3.7 (2.8–4.5)
AFP (ng/mL)	5215 (2.8–48 000)
AFP‐L3% (%)	36.4 (0–91.3)
DCP (mAU/mL)	1318 (12–9399)

† As of the last follow‐up date: 31 August 2013.

AFP, alpha‐fetoprotein; AFP‐L3%, *Lens culinaris* agglutinin‐reactive fraction of AFP; ALT, alanine aminotransferase; AST, aspartate aminotransferase; BMI, body mass index; DCP, Des‐gamma‐carboxy prothrombin; ECOG PS, Eastern Cooperative Oncology Group performance status; HBV, hepatitis B virus; HCV, hepatitis C virus; NBNC, nonhepatitis B nonhepatitis C.

### *Survival analyses*

**Figure 1:**
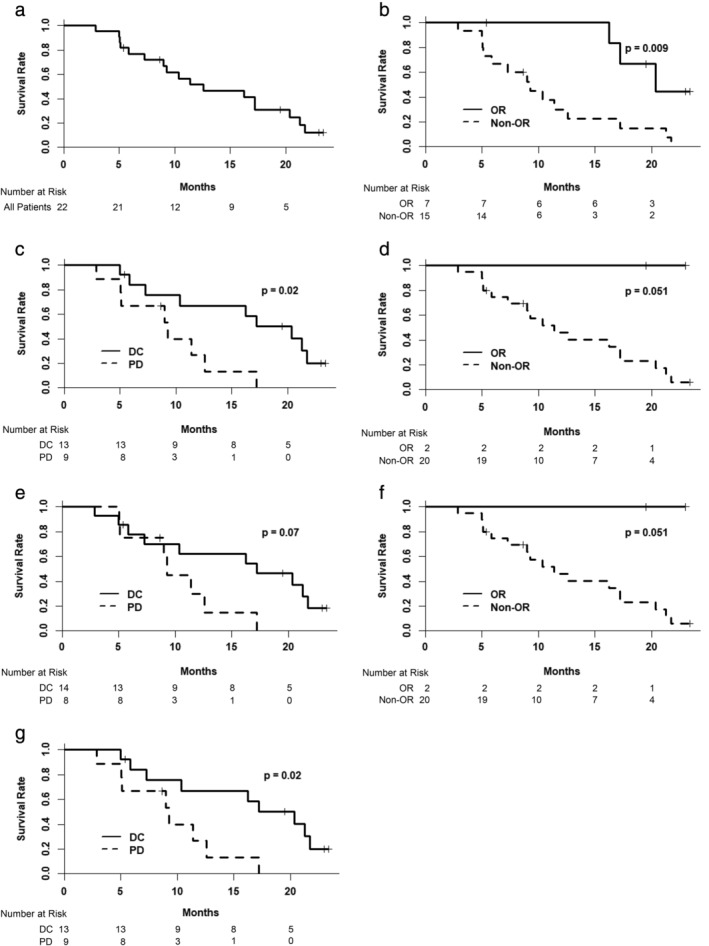
Comparison of Kaplan–Meier analyses of overall survival. (a) All patients (*n* = 22); (b) volume and density criteria: OR *versus* non‐OR; (c) volume and density criteria: DC *versus* PD; (d) RECIST 1.1: OR *versus* non‐OR; (e) RECIST 1.1: DC *versus* PD; (f) volumetric criteria: OR *versus* non‐OR; and (g) volumetric criteria: DC *versus* PD. DC, disease control; OR, objective response; PD, progressive disease; RECIST, Response Evaluation Criteria in Solid Tumors.

The median OS was 12.6 months for all patients (95% confidence interval [CI], 9.0–21.2; Fig. 1a). A total of 44 follow‐up time points were reviewed for radiological assessment of treatment responses. When patients were dichotomized into OR and non‐OR groups according to volume and density criteria, the median OS was 20.4 months for the OR group and 9.3 months for the non‐OR group (Fig. 1b, *P* = 0.009). When the patients were dichotomized into DC and PD groups according to volume and density criteria, the median OS was 20.4 months for the DC group and 9.3 months for the PD group (Fig. 1c, *P* = 0.02). When patients were dichotomized into OR and non‐OR groups according to RECIST 1.1, the median OS for the non‐OR group was 11.4 months (Fig. 1d, *P* = 0.051; the median OS was not reached by the OR group). When patients were dichotomized into DC and PD groups according to RECIST 1.1, the median OS was 17.2 months for the DC group and 9.3 months for the PD group (Fig. 1e, *P* = 0.07). When patients were dichotomized into OR and non‐OR groups according to volumetric criteria, the median OS for the non‐OR group was 11.4 months (Fig. 1f, *P* = 0.051; the median OS was not reached by the OR group). When the patients were dichotomized into DC and PD groups according to volumetric criteria, the median OS was 20.4 months for the DC group and 9.3 months for the PD group (Fig. 1g, *P* = 0.02). The Kaplan–Meier analyses by mRECIST are available in Figure S1, Supporting information. Median OS was 10.4 months for the OR group and 12.6 months for the non‐OR group according to mRECIST (*P* = 0.58). The median OS was 18.8 months for the DC group and 9.0 months for the PD group according to mRECIST (*P* = 0.09).

Classification of tumor responses according to the four criteria is summarized in Table 3. DC rates were lower for patients dichotomized according to mRECIST than they were for those assessed using other criteria (40.9% for mRECIST; 63.6% for RECIST 1.1; 59.1% for both volumetric criteria and volume and density criteria, chi‐squared test, *P* = 0.44). In contrast, the OR rate was higher for volume and density criteria than for other criteria (31.8% for volume and density criteria *vs* 9.1% for other criteria, chi‐squared test, *P* = 0.08). All reclassifications based on volume and density criteria, as opposed to RECIST 1.1 criteria, were observed as changes from SD to PD or PR groups. Most of these (five of six patients) were classified as better responders according to volume and density criteria. Similarly, volume and density criteria reclassified many cases (10 of 12 patients) as better responders compared to mRECIST.

**Table 3:** Classification of tumor responses according to three evaluation criteria

	CR	PR	SD	PD
	DC			PD
	OR		Non‐OR	
RECIST 1.1	1	1	12	8
Volumetry	1	1	11	9
Volume and density	1	6	6	9
mRECIST	1	1	7	13

CR, complete response; DC, disease control; OR, objective response; PD, progressive disease; PR, partial response; SD, stable disease; RECIST, Response Evaluation Criteria in Solid Tumors; mRECIST, modified RECIST.

### *Importance of different variables and selection of the best variables for predicting survival*

First, the importance of both pretreatment and peritreatment variables was measured using the random forest approach for survival outcome. Pretreatment variables included the LD of the hepatic lesions, extrahepatic spread (i.e. metastasis: Yes or No), vascular invasion (Yes or No), lymph node lesions (Yes or No), age, smoking history (Yes or No), alcohol consumption (Yes or No), AFP level, and DCP level. Peritreatment variables included RECIST 1.1 OR (OR or non‐OR), RECIST 1.1 DC (DC or PD), volumetric criteria OR (OR or non‐OR), volumetric criteria DC (DC or PD), volume and density criteria OR (OR or non‐OR), volume and density criteria DC (DC or PD), mRECIST OR (OR or non‐OR), mRECIST DC (DC or PD), and the finding of a new lesion (Yes or No). This random forest‐based analysis identified the following variables as having relatively high importance (these were then entered into the Cox regression analysis): RECIST 1.1 OR, RECIST 1.1 DC, volumetric criteria OR, volumetric criteria DC, volume and density criteria OR, volume and density criteria DC, mRECIST DC, extrahepatic spread, AFP level, and smoking history (Table 4).

**Table 4:** Importance of variables (identified using the random forest approach)

Variable	Importance
Pretreatment variables	
Age	−2.78
Longest diameter of hepatic lesions	−0.78
Extrahepatic spread	3.65
Vascular invasion	−0.99
Lymph node lesion	−0.03
AFP level	3.23
DCP level	−1.38
Smoking history	1.96
Alcohol consumption	−1.04
Peritreatment variables	
RECIST 1.1 OR	2.58
RECIST 1.1 DC	1.82
Volumetric criteria OR	1.41
Volumetric criteria DC	2.88
Volume and density criteria OR	9.66
Volume and density criteria DC	5.16
mRECIST OR	0.84
mRECIST DC	6.14
New lesion	−2.27

Variables with a large positive importance value were considered important. The actual value may vary according to the random seed used.

AFP, alpha‐fetoprotein; DC, disease control; DCP, Des‐gamma‐carboxy prothrombin; OR, objective response; RECIST, Response Evaluation Criteria in Solid Tumors; mRECIST, modified RECIST.

Next, univariate Cox regression analyses were conducted using the important variables identified by the random forest approach described above. The log‐rank test‐based selection of prognostic variables identified RECIST 1.1 DC (*P* = 0.07), volumetric criteria DC (*P* = 0.02), volume and density criteria OR (*P* = 0.01), volume and density criteria DC (*P* = 0.02), mRECIST DC (*P* = 0.09), and AFP level (*P* = 0.09) as prognostic variables (Table 5). Finally, AIC‐based selection demonstrated that the best multivariate regression model had two significant variables: volume and density criteria OR (hazard ratio [HR], 5.4; 95% CI, 1.5–20.0) and AFP level (HR, 1.5; 95% CI, 1.0–2.2) (log‐rank test, *P* = 0.01) (Table 6).

**Table 5:** Results of univariate Cox regression analysis

Variable	*P* value†	AIC	Hazard ratio	95% CI	*P* value
RECIST 1.1					
OR/non‐OR	0.05	76.00	—	—	—
RECIST 1.1					
DC/PD	0.07	79.78	2.65	0.89–7.85	0.08
Volumetric criteria					
OR/non‐OR	0.05	76.00	—	—	—
Volumetric criteria					
DC/PD	0.02	77.64	3.56	1.18–10.7	0.02
Volume and density criteria					
OR/non‐OR	0.01	75.26	4.75	1.34–16.9	0.02
Volume and density criteria					
DC/PD	0.02	77.64	3.56	1.18–10.7	0.02
mRECIST					
DC/PD	0.09	79.86	2.35	0.86–6.45	0.10
AFP level	0.09	80.00	1.40	0.95–2.06	0.09
Smoking history					
Yes/no	0.19	81.05	1.94	0.71–5.31	0.20
Extrahepatic spread					
Yes/no	0.17	81.07	0.51	0.19–1.37	0.18

† Log‐rank test.

—, not available; AFP, alpha‐fetoprotein; AIC, Akaike's Information Criterion; CI, confidence interval; DC, disease control; OR, objective response; PD, progressive disease; RECIST, Response Evaluation Criteria in Solid Tumors; mRECIST, modified RECIST.

**Table 6:** Results of multivariate Cox regression analysis

Variable	*P* value†	AIC	Hazard ratio	95% CI	*P* value
RECIST 1.1 DC	0.07	79.97	2.30	0.74–7.14	0.15
AFP level			1.30	0.89–1.91	0.18
Volumetric DC	0.02	78.11	3.16	1.01–9.93	0.049
AFP level			1.27	0.87–1.85	0.22
Volume and density criteria OR	0.01	73.51	5.45	1.48–20.0	0.01
AFP level			1.47	1.00–2.17	0.0497
Volume and density criteria DC	0.02	78.11	3.16	1.01–9.93	0.049
AFP level			1.27	0.87–1.85	0.22
mRECIST DC	0.12	80.71	1.88	0.63–5.66	0.26
AFP level			1.26	0.83–1.91	0.29

† Log‐rank test.

AFP, alpha‐fetoprotein; AIC, Akaike's Information Criterion; CI, confidence interval; DC, disease control; OR, objective response; RECIST, Response Evaluation Criteria in Solid Tumors; mRECIST, modified RECIST.

## Discussion

RECIST 1.1‐based radiological assessment is used widely for treatment response classification and as a surrogate end‐point both in clinical trials and in routine practice. However, the development of molecular targeted therapies to lessen tumor vascularity has shown that the RECIST 1.1 assessment has limitations.^7^, ^9^, ^17^, ^21^ Some modifications to RECIST 1.1 for HCC treatment have been proposed, including mRECIST, EASL, and the Choi criteria, which are considered to better reflect treatment effects.^9^ However, these modifications are still based on a unidimensional manual assessment and are strongly affected by heterogeneities in tumor appearance, that is, CT enhancement pattern, induced by sorafenib and other locoregional therapies such as radiofrequency ablation (RFA) and transcatheter arterial chemoembolization (TACE). In daily clinical settings, we often encounter a variety of changes in tumor shape and vascularity. Therefore, a reproducible and objective method, such as the use of automated computer‐assisted volume and density measurement that captures therapeutic changes in the whole lesion, is desirable. For example, two of the cases in this study showed much longer survival times (16.2 and 20.4 months) than expected according to RECIST 1.1 criteria (Fig. S2). When we applied the new volume and density criteria to these patients, their evaluation changed to PR rather than the PD or SD evaluation obtained using RECIST 1.1. This evaluation was more acceptable as a radiological assessment of treatment response because it correlated well with OS and was reproducible. Thus, to overcome the many limitations of conventional criteria in routine practice, we examined new radiological assessment methods based on the automated measurement of volume and density changes in hepatic lesions on CT scans. The aim was to achieve a simpler and more accurate classification of prognostic responses than those obtained using unidimensional or three‐dimensional volumetric measurements alone. The main point is that this new method considers both morphological (volume) and functional (density) aspects of the lesions simultaneously and automatically. As a consequence, it enables more appropriate discrimination of good responders from SD patients. In comparison, mRECIST identified equal or fewer responders compared to other response evaluation criteria. The DC rate was 40.9% (*vs* volume and density criteria, *P* = 0.23), and the OR rate was 9.1% (*vs*. volume and density criteria, *P* = 0.07). The routine use of mRECIST has limitations because irregular morphological changes of tumor enhancement could not control its accuracy of objective unidimensional measurement of a viable part by assessors. In contrast, new volume and density criteria were further confirmed as good classifiers using several statistical models, including the random forest approach and Cox regression analysis. Even though the results are subject to the limitations discussed below, the automated volume and density criteria approach appears to be a superior objective method of radiological assessment of the effects of sorafenib treatment. The new method may also offer better prediction of OS because it can globally reflect both the shape and vascularity (the major parameters affected by sorafenib) of the lesion during/after treatment with sorafenib.

In addition to the radiological/imaging factors, the random forest approach and Cox regression analysis identified the prognostic potential of baseline AFP levels, although the results of Kaplan–Meier analysis did not show a statistically significant classification of the patients according to AFP level; this is due to the small sample size (log‐rank test between ≥400 ng/mL [*n* = 10] and <400 ng/mL [*n* = 12], *P* = 0.14). AFP is an established tumor marker for HCC and may be associated with the prognosis of HCC patients. Several studies have proposed AFP as a marker that can be used to assess HCC responses to targeted chemotherapy because of its ability to discriminate patients with longer OS.^22^, ^23^, ^24^ Consequently, further studies should investigate the ability of combined radiological assessment plus AFP levels to predict OS.

An automated and objective assessment of treatment response, such as that demonstrated here, could help to establish a cloud computer system for data collection or a clinical data repository for HCC therapy. This would facilitate “big data” applications and allow global multisite clinical trials to be conducted efficiently.

This study has several limitations. First, the study was retrospective in design, with a small number of patients from a limited area of Japan. Real‐world cases often present difficulties with respect to precise radiological assessment due to major morphological modifications caused by other prior locoregional treatments. This may lead to structural uncertainty when determining cut‐off values for the classification of treatment responses. Based on our results from a limited patient cohort, further studies should be conducted to establish robust cut‐off values for volume and density changes. Second, the impact of reproducibility on assessments made by automated software measurements was not examined in detail. For the objective measurement of lesion volume and density, the assistance of computer software is both critical and inevitable. Therefore, a software validation study is also required for this scheme. Third, the acquisition protocol for CT scans may affect the radiological assessment through changes in image quality, particularly with respect to density measurements on the arterial phase acquisition. The CT scan images in this study were obtained from two neighboring hospitals, but the acquisition protocol may vary across other institutions; thus, operationally acceptable guidelines for imaging quality control are necessary for the global expansion of our method.

This exploratory study suggests that the automated measurement of tumor volume and density on CT scans using computer software could be a better method of assessing responses of patients with advanced HCC to treatment with sorafenib. This radiological assessment method was good at reflecting survival outcomes. Concerns raised about the routine use of RECIST 1.1 to assess tumor responses to sorafenib therapy could be addressed using this software‐based standardized approach to three‐dimensional radiological assessment.

## Supporting information


**Figure S1** Kaplan–Meier analyses of overall survival based on mRECIST. (a) OR *versus* non‐OR; (b) DC *versus* PD. DC, disease control; OR, objective response; PD, progressive disease; mRECIST, modified Response Evaluation Criteria in Solid Tumors.

**Figure S2** Representative findings of individual patients. (a) An 82‐year‐old male (HBV[+], ECOG PS 0 grade, Child‐Pugh 6 points); OS = 16.2 months; PD (RECIST 1.1), PR (volume and density criteria). (b) A 73‐year‐old male (HCV[+], ECOG PS grade 0, Child‐Pugh 5 points); OS = 20.4 months; SD (RECIST 1.1), PR (volume and density criteria). ECOG PS, Eastern Cooperative Oncology Group performance status; HBV, hepatitis B virus; HCV, hepatitis C virus; PD, progressive disease; PR, partial response; SD, stable disease; RECIST, Response Evaluation Criteria in Solid Tumors.
