# Supplementary figures and images for: Assessment of tumor volume and density as a measure of the response of advanced hepatocellular carcinoma to sorafenib: Application of automated measurements on computed tomography scans
Source: JGH Open. 2019 Aug 2;4(2):145–52. doi: 10.1002/jgh3.12230 (PMC7144795; doi:10.1002/jgh3.12230)

**a**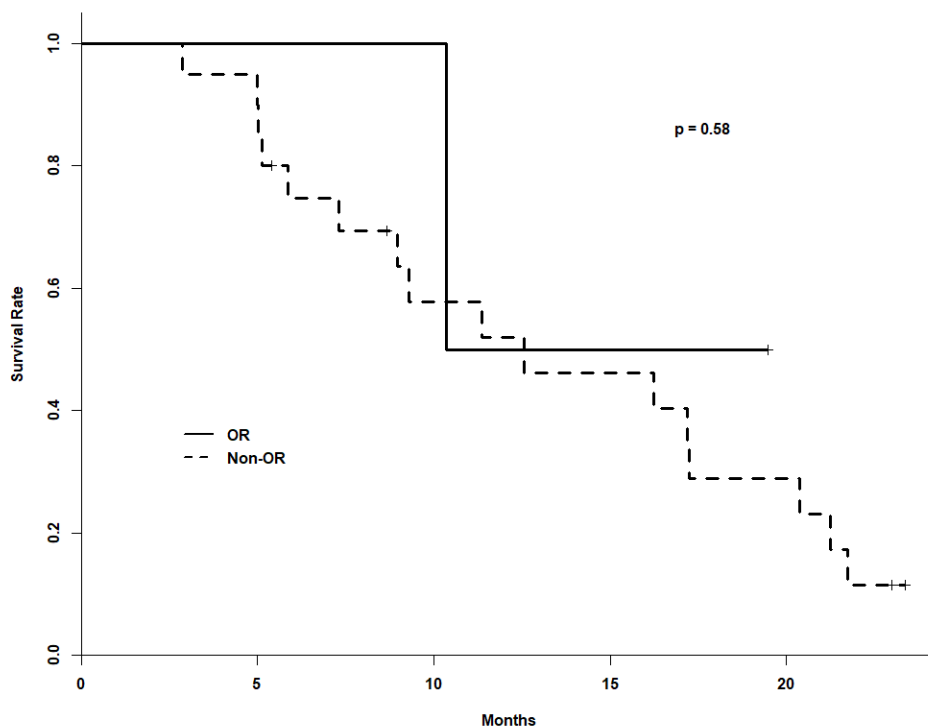

Number at Risk

|        |    |    |    |   |   |
|--------|----|----|----|---|---|
| OR     | 2  | 2  | 2  | 1 | 0 |
| Non-OR | 20 | 19 | 10 | 8 | 5 |

**b**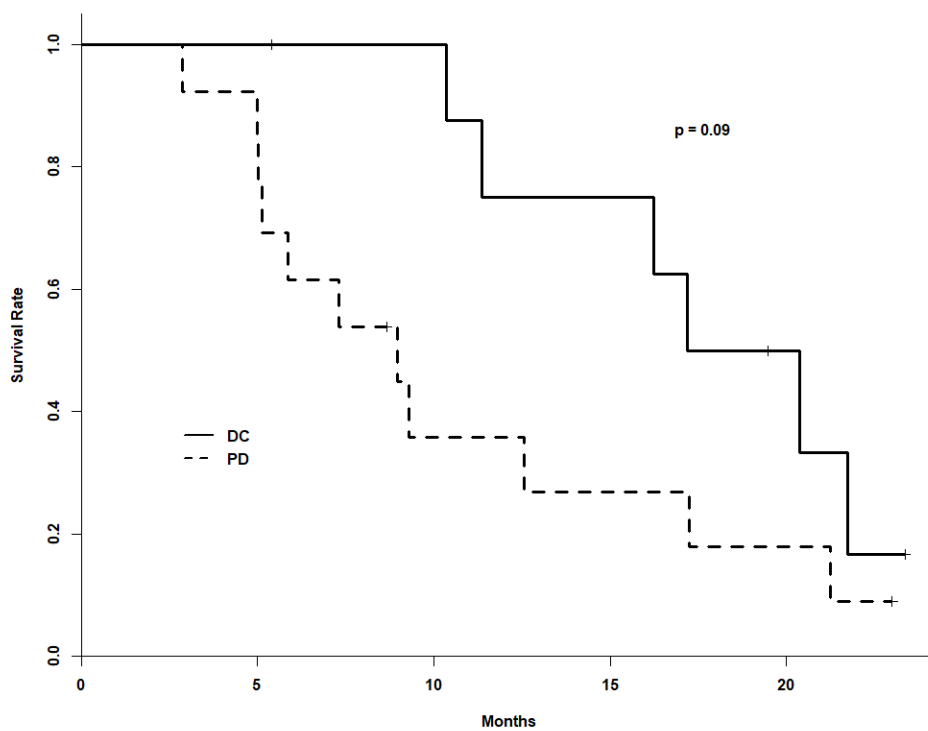

Number at Risk

|    |    |    |   |   |   |
|----|----|----|---|---|---|
| DC | 9  | 9  | 8 | 6 | 3 |
| PD | 13 | 12 | 4 | 3 | 2 |

Supplement: Supplementary file 1 — Figure S1 Kaplan–Meier analyses of overall survival based on mRECIST. (a) OR versus non‐OR; (b) DC versus PD. DC, disease control; OR, objective response; PD, progressive disease; mRECIST, modified Response Evaluation Criteria in Solid Tumors. [file JGH3-4-145-s001.pdf]

a

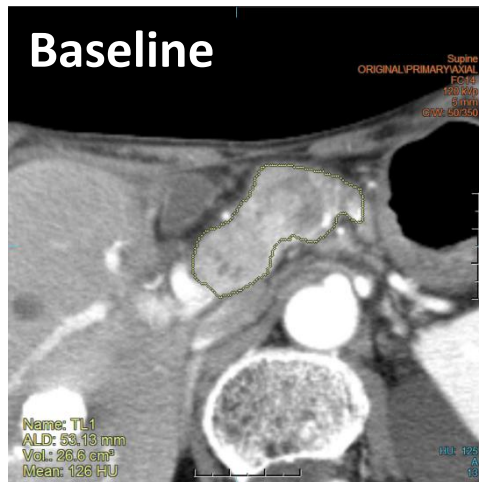**After 3 months**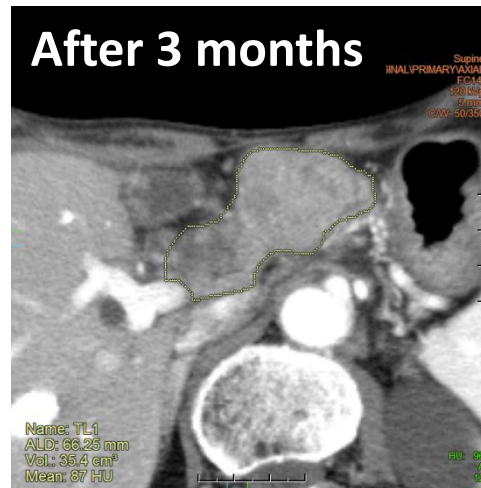

b

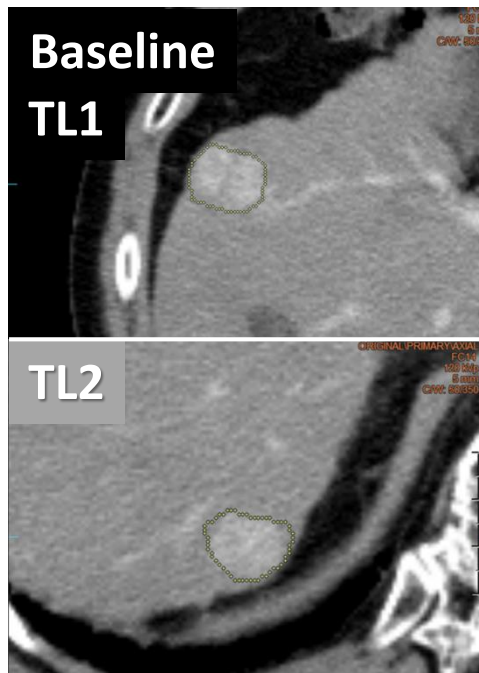**After 3 months**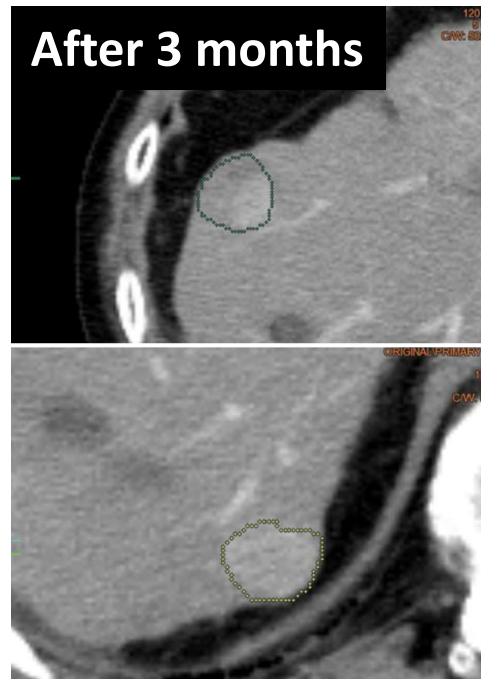

Supplement: Supplementary file 2 — Figure S2 Representative findings of individual patients. (a) An 82‐year‐old male (HBV[+], ECOG PS 0 grade, Child‐Pugh 6 points); OS = 16.2 months; PD (RECIST 1.1), PR (volume and density criteria). (b) A 73‐year‐old male (HCV[+], ECOG PS grade 0, Child‐Pugh 5 points); OS = 20.4 months; SD (RECIST 1.1), PR (volume and density criteria). ECOG PS, Eastern Cooperative Oncology Group performance status; HBV, hepatitis B virus; HCV, hepatitis C virus; PD, progressive disease; PR, partial response; SD, stable disease; RECIST, Response Evaluation Criteria in Solid Tumors. [file JGH3-4-145-s002.pdf]
